# Recent Advances in the Total Synthesis of Spirotryprostatin Alkaloids

**DOI:** 10.3390/molecules29071655

**Published:** 2024-04-07

**Authors:** Jing Hu, Zhen-Xi Niu, Jun-Feng Wang

**Affiliations:** 1 Children’s Hospital Affiliated to Zhengzhou University, Henan Children’s Hospital, Zhengzhou Children’s Hospital, Zhengzhou 450018, China; 2 Gordon Center for Medical Imaging, Massachusetts General Hospital and Harvard Medical School, 125 Nashua Street, Suite 660, Boston, MA 02114, USA

**Keywords:** spirotryprostatin, total synthesis, spiro-C atom, chirality control

## Abstract

Spirotryprostatin alkaloids, a class of alkaloids with a unique spirocyclic indoledionepiperazine structure, were first extracted from the fermentation broth of Aspergillus fumigatus and have garnered significant attention in the fields of biology and pharmacology. The investigation into the pharmacological potential of this class of alkaloids has unveiled promising applications in drug discovery and development. Notably, certain spirotryprostatin alkaloids have demonstrated remarkable anti-cancer activity, positioning them as potential candidates for anti-tumor drug development. In recent years, organic synthetic chemists have dedicated efforts to devise efficient and viable strategies for the total synthesis of spirotryprostatin alkaloids, aiming to meet the demands within the pharmaceutical domain. The construction of the spiro-C atom within the spirotryprostatin scaffold and the chirality control at the spiro atomic center emerge as pivotal aspects in the synthesis of these compounds. This review categorically delineates the synthesis of spirotryprostatin alkaloids based on the formation mechanism of the spiro-C atom.

## 1. Introduction

**Scheme 1:**
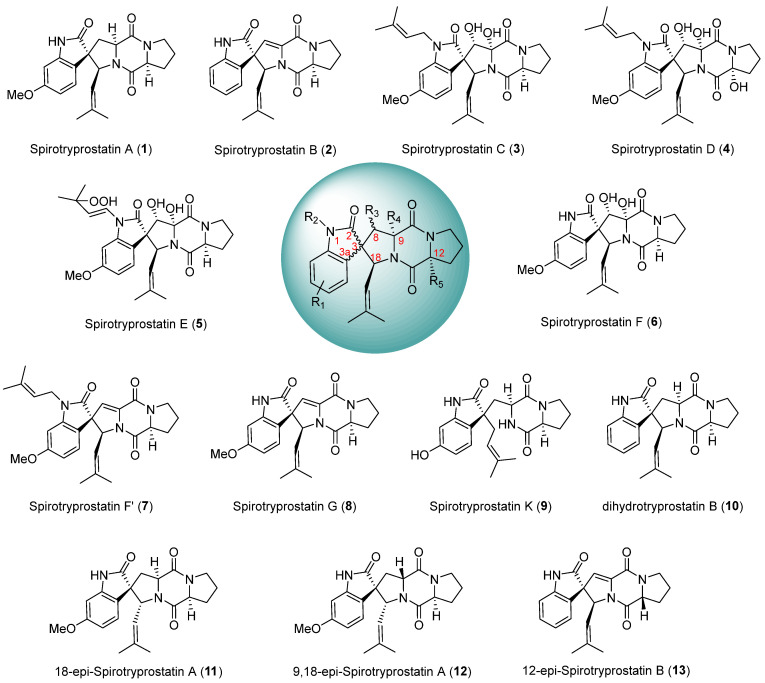
Main structures and diastereomers of spirotryprostatin alkaloids.

The spirotryprostatin class of alkaloids represents a significant category of spiroindolinone-piperazine alkaloids, characterized by a fundamental pentacyclic structure composed of a spiroindolinone moiety and a dioxopiperazine moiety, featuring multiple chiral centers. Initially isolated by Osada et al. [1,2] from the fermentation broth of Aspergillus fumigatus, two distinct spirotryprostatin alkaloids were first identified and designated spirotryprostatin A (**1**) and spirotryprostatin B (**2**). In the course of activity assays, it was observed that spirotryprostatins A and B exhibit inhibitory effects on murine breast cancer cells. Furthermore, their cytotoxic activity against human chronic myeloid leukemia cells and human acute promyelocytic leukemia cells was confirmed, suggesting the potential of spirotryprostatin-class alkaloids as lead compounds for novel drug screening endeavors [3,4,5,6]. This underscores the prospect of spirotryprostatins being promising candidates in the identification of novel therapeutic agents. The spiroindolinone and dioxopiperazine motifs within the structural framework of spirotryprostatin-class alkaloids represent pivotal elements found in numerous synthetic and natural compounds [7,8,9,10]. Compounds featuring the spiroindolinone moiety have demonstrated diverse biological activities [11,12,13], including anti-tumor, antimicrobial, anti-HIV, and anti-malarial properties. Similarly, those containing the dioxopiperazine moiety exhibit bioactivities [14,15,16] such as cytotoxicity, antimicrobial effects, antifungal properties, and thrombosis prevention. Notably, spirotryprostatin-class alkaloids uniquely combine both of these critical motifs, thereby manifesting significant and versatile biological activities. As of the present moment, ten principal structures of spirotryprostatin alkaloids [17,18,19,20,21] have been isolated (Scheme 1), and their pharmacological activities [22,23,24], including anti-tumor, anti-HIV, insecticidal [24], and analgesic effects have been substantiated. Consequently, these compounds have garnered extensive attention from the chemical community. However, it is noteworthy that spiroindolinone compounds, despite their promising pharmacological profiles, remain at the cellular experimentation stage in clinical research [25,26], with no current reports documenting their progression into marketable products.

Since the discovery of spirotryprostatin-class alkaloids, numerous research groups have undertaken diverse synthetic approaches to their total synthesis (Table 1). The scope of these synthetic endeavors extends beyond the original spirotryprostatin alkaloids isolated and encompasses the total synthesis of modified derivatives [15,27,28,29,30,31,32,33,34,35]. Through structural modifications, the objective is not only to recreate the originally isolated spirotryprostatin alkaloids but also to generate a novel cohort of spirotryprostatin-class alkaloids with enhanced pharmacological efficacy. This synthetic modification approach aims to yield new pharmaceutical agents within the spirotryprostatin class with augmented therapeutic potential. The construction of the spiro-C atom within the spirotryprostatin scaffold and the chiral control at the spiro atomic center are pivotal in the synthesis of such compounds. The spiro-C atom located at the indole nucleus’s C3 position serves as the chiral center for the entire molecular framework, formed by the shared carbon atom between the oxidized indole and the pyrrolidine. Owing to the stereochemical selectivity inherent in chemical reactions, the total synthesis of certain analogous compounds often yields diastereoisomers of the target product (Scheme 1). In summary, the synthesis of compounds of this nature encounters challenges primarily in the construction of quaternary carbon atoms and the chiral control at the spiro-C center. Current synthetic methodologies for such compounds predominantly involve the rational design of substrates functionalized at the C3 position of the indole moiety, followed by ring-closure processes to form the spiro-indole-pyrrolidine framework. This review categorically reports the synthesis of spirotryprostatin-class alkaloids based on the manner in which the spiro-C atom is formed.

## 2. The Synthetic Approach Employing Tryptophan Derivatives as the Starting Material

### 2.1. The Oxidative Rearrangement Methodology

#### 2.1.1. Danishefsky’s Total Synthesis

**Scheme 2:**
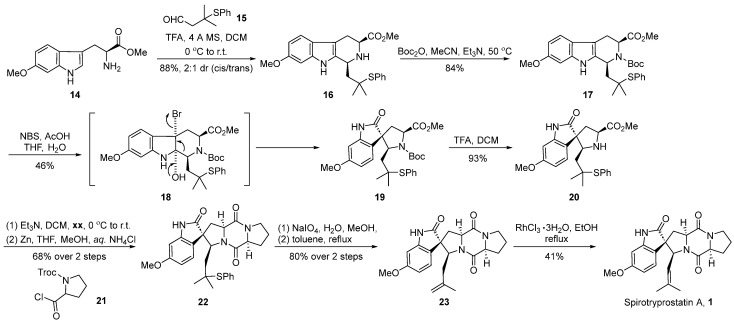
Danishefsky’s total synthesis of spirotryprostatin A via oxidative rearrangement.

In 1998, Danishefsky’s group [36] first reported the total synthesis pathway of spirotryprostatin A. Subsequently, they modified the reaction pathway, successfully achieving the total synthesis of spirotryprostatin A (**1**) and its derivative dihydrotryprostatin B (**10**) (Scheme 2). A pivotal step in the reaction involved the oxidative rearrangement of a β-carboline derivative facilitated by NBS, leading to the formation of the spirocyclic moiety. The reaction commenced with 6-methoxytryptophan methyl ester **14** and tert-butylthioacetaldehyde **15** as starting materials. Initially, subjected to a Pictet–Spengler cyclization reaction, the process yielded the indole and hexacyclic intermediate **16** with a diastereomeric ratio of 2:1. The subsequent protection of the amino group with a Boc moiety led to the formation of intermediate **17**. Furthermore, under the influence of NBS and acetic acid, an oxidative rearrangement ensued, culminating in the formation of the crucial pyrrolidine-indole ketone spirocyclic intermediate **19**. Subsequently, intermediate **19** underwent deprotection facilitated by trifluoroacetic acid, followed by a two-step condensation with proline derivative **21** to form the dioxopiperazine structural intermediate **22**. Finally, intermediate **22** underwent oxidation with sodium periodate, followed by the elimination of the phenylthioether to generate terminal alkene **23**. Under the catalysis of rhodium trichloride hydrate, the double bond underwent isomerization, resulting in the target product spirotryprostatin A (**1**). The overall synthesis spanned eight steps with a total yield of 6.5%.

#### 2.1.2. Granesan’s Total Synthesis

**Scheme 3:**
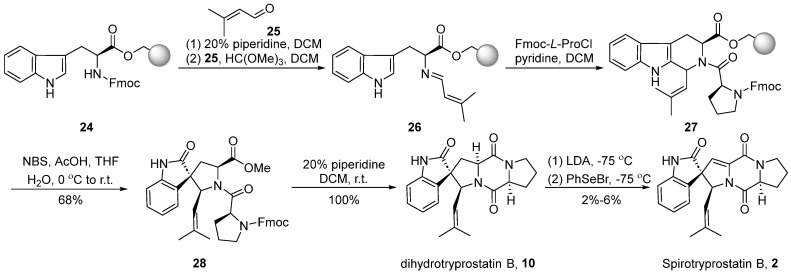
Granesan’s total synthesis of spirotryprostatin B via oxidative rearrangement.

Around the year 2000, Granesan’s group [37,38,39] applied the PhSeBr reagent elimination reaction to the dioxopiperazine system for the construction of double bonds. Simultaneously, oxidative rearrangement was employed as a crucial step in spirocyclic formation. This strategic approach led to the successful total synthesis of dihydrospirotryprostatin B (**10**) and spirotryprostatin B (**2**). In the initial stages of Granesan’s synthesis, the traditional starting point still involved *L*-tryptophan methyl ester. However, in the optimized approach, the starting material was modified to utilize *L*-tryptophan **24** protected with *N*-Fmoc and anchored to polystyrene resin [38] (Scheme 3). In the presence of piperidine, the Fmoc protecting group was removed from the starting material, liberating the amino group. This amino group then reacted with isovaleraldehyde **25** and methyl formate to form an imine intermediate **26**. Subsequently, imine **26** underwent a Pictet-Spengler reaction with *N*-Fmoc-*L*-proline acyl chloride, leading to the formation of dipeptide intermediate **27** with a cis/trans ratio of 1:1 at the C18 position. Intermediate **27**, under the influence of NBS and acetic acid, underwent an oxidative rearrangement to yield the spirocyclic intermediate **28**. The subsequent removal of the Fmoc protecting group from the proline moiety, followed by a ring-closure process, led to the formation of dihydrospirotryprostatin B (**10**). The latter underwent a PhSeBr-mediated elimination reaction to yield the final product spirotryprostatin B (**2**). The entire reaction sequence encompassed five steps with an overall yield ranging from 2–6%, accompanied by the generation of several byproducts.

#### 2.1.3. Zhang’s Total Synthesis

**Scheme 4:**
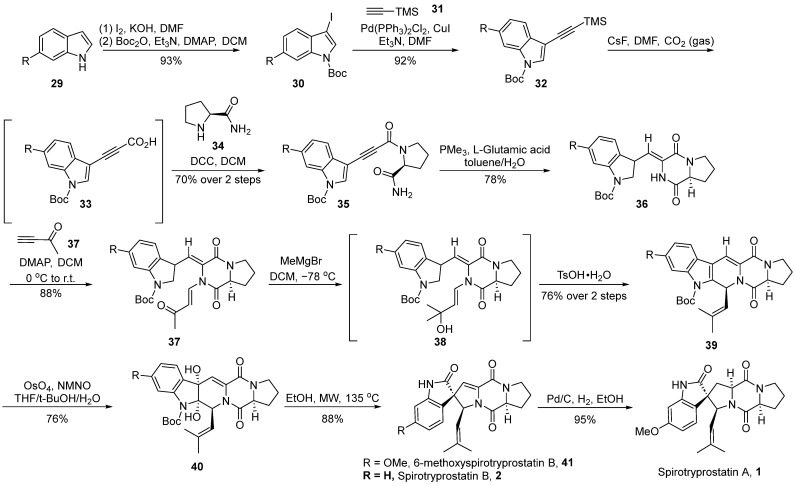
Zhang’s total synthesis of spirotryprostatin alkaloids via OsO_4_-mediated oxidative rearrangement.

In 2019, Zhang et al. [40] reported the total synthesis of spirotryprostatin A (**1**), achieved through intramolecular cyclization and osmium tetroxide-mediated oxidative rearrangement processes (Scheme 4). The authors initiated the synthesis using 6-substituted indole **29** as the starting material, which underwent iodination followed by Boc protection, resulting in *N*-Boc-iodinated indole compound **30**. Compound **30** was then subjected to Sonogashira coupling with trimethylsilylacetylene **31**, yielding the alkyne-silane intermediate **32**. Alkyne-silane **32** underwent carboxylation with CO_2_, forming unstable intermediate **33**. Subsequently, direct condensation with (S)-prolyl amide **34** led to the formation of alkyne amide **35**. Alkyne amide **35** underwent intramolecular cyclization to generate dioxopiperazine intermediate **36** with E/Z = 7:1. Subsequent nucleophilic addition with 3-buten-2-one **37**, followed by further addition with a Grignard reagent, resulted in tertiary alcohol intermediate **38**. The dehydration of intermediate **38**, catalyzed by TsOH, formed a double bond, which, upon selective oxidation with osmium tetroxide, led to the formation of diol compound **40**. Subsequently, compound **40** underwent rearrangement under high-temperature microwave conditions. When the R-group was hydrogen, spirotryprostatin B (**2**) was directly obtained, whereas the use of methoxy-substituted indole as the starting material yielded 6-methoxyspirotryprostatin B **41**. The latter, upon the Pd/C-catalyzed reduction of the double bond, afforded the natural product spirotryprostatin A (**1**). The entire reaction sequence comprised 11 steps, resulting in a 20% overall yield.

### 2.2. Mannich Reaction

#### Danishefsky’s Total Synthesis

**Scheme 5:**
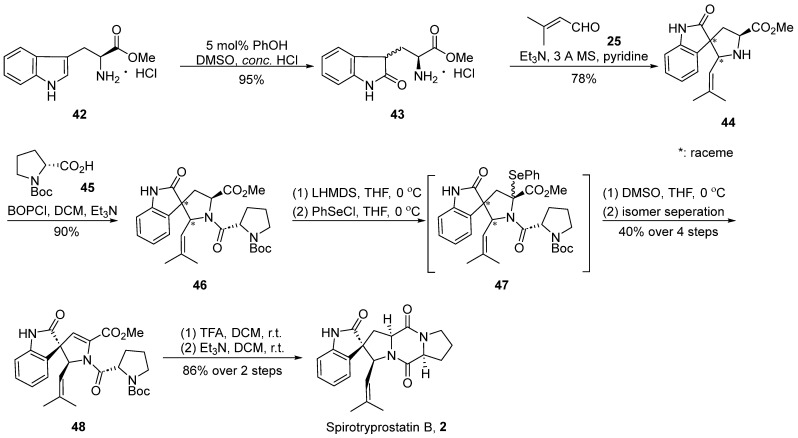
Danishefsky’s total synthesis of spirotryprostatin B via Mannich reaction.

In the year 2000, Danishefsky’s group [41] accomplished the inaugural total synthesis of spirotryprostatin B (**2**), employing the Mannich reaction as a pivotal step in the formation of the spirocyclic moiety (Scheme 5). The reaction commenced with *L*-tryptophan methyl ester hydrochloride **42** and, under the influence of phenol and concentrated hydrochloric acid, underwent transformation into the oxidized indole structure compound **43**. Subsequently, through a Mannich reaction with 3-buten-2-one **25**, four distinct spirocyclic indole ketone intermediate-like compounds **44**, each possessing an isobutene side chain, were obtained. Without the isolation of the four isomers, they were directly condensed with Boc-protected proline **45**, yielding a mixture of compound **46**. In the final step, a method involving the elimination of PhSeCl reagent under the influence of LHMDS was employed, leading to the introduction of a double bond at positions C8 and C9. The reaction yielded a mixture of isomers, from which a singular isomer **48** was isolated. Following the removal of protective groups using TFA and a subsequent cyclization step catalyzed by triethylamine, the target compound spirotryprostatin B (**2**) was obtained. The entire reaction sequence encompassed eight steps, resulting in an overall yield of 4.6%.

The comprehensive synthesis strategy involved the Mannich reaction between an *L*-tryptophan derivative and an aldehyde to construct the envisaged spirocyclic indole scaffold. While the desired product was successfully obtained, the approach introduced significant stereochemical challenges, inevitably resulting in a mixture of diastereomers. Despite subsequent attempts to assist the separation of diastereomeric mixtures using *L*-Boc-protected proline in later reactions, the overall yield of spirotryprostatin B was notably diminished.

### 2.3. Intramolecular N-Acyliminium Ion Spirocyclic Cyclization

#### Horne’s Total Synthesis

**Scheme 6:**
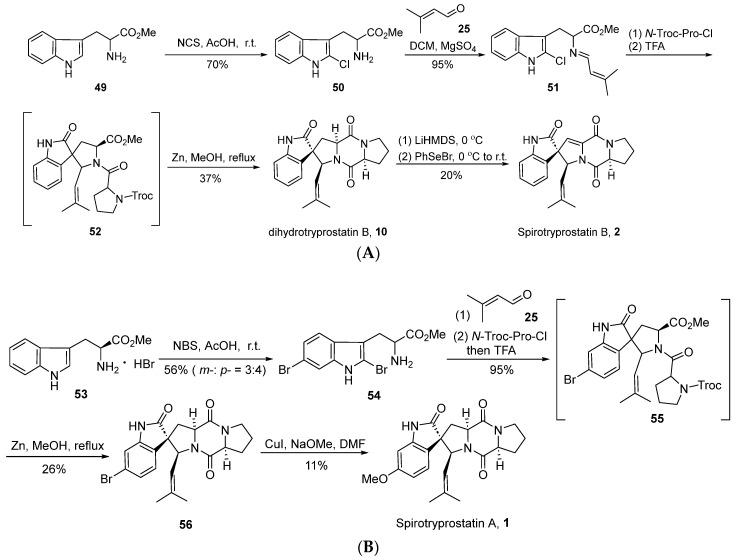
(**A**) Horne’s total synthesis of spirotryprostatin B via intramolecular *N*-acyliminium ion spirocyclic cyclization. (**B**) Horne’s total synthesis of spirotryprostatin A via intramolecular *N*-acyliminium ion spirocyclic cyclization.

In 2004, Horne’s group [42] successfully achieved the total synthesis of spirotryprostatin B (**2**) through an innovative strategy based on intramolecular *N*-acyliminium ion spirocyclic cyclization, utilizing 2-halotryptophan esters as key intermediates (Scheme 6A). The reaction initiated with *L*-tryptophan methyl ester **49**, which underwent a reaction with *N*-chlorosuccinimide (NCS) to yield 2-chlorotryptophan methyl ester **50**. Subsequent condensation with isovaleraldehyde **25** resulted in the formation of imine intermediate **51**. Under the influence of *N*-Troc-protected proline acyl chloride and trifluoroacetic acid, intermediate **51** underwent acylation, inducing spirocyclic cyclization and yielding indole-oxidized spirocyclic intermediate **52**. In contrast to the conventional oxidative rearrangement reactions, a distinctive feature of this reaction lies in the hindrance of the indole’s 2-position by halogen, leading to electrophilic attack at the 3-position and subsequent spirocyclic formation. Subsequently, under the influence of zinc, the Troc-group underwent departure, yielding dihydrospirotryprostatin B and three distinct isomers. Finally, the authors employed a non-oxidative method to convert dihydrospirotryprostatin B (**10**) into spirotryprostatin B (**2**), involving a seven-step reaction sequence with an overall yield of 4.9%. This route innovatively utilized the strategy of *N*-acyliminium ion spirocyclic cyclization for constructing the oxidized indole spirocyclic moiety. However, the lack of control over enantioselectivity in this approach, coupled with the generation of relevant byproducts, limits the applicability of this route.

In the same year, Horne et al. [43] successfully achieved the total synthesis of spirotryprostatin A (**1**) using a similar methodology (Scheme 6B). The route commenced with *L*-tryptophan methyl ester **53**, wherein excess NBS was employed for bromination. However, this step exhibited limited regioselectivity, leading to bromination at both the C5 and C6 positions. Subsequently, employing a strategy analogous to the synthesis of spirotryprostatin B, intermediate compound **56** was obtained. Through a copper-catalyzed methoxylation strategy, the bromine moieties were converted into methoxy groups, culminating in the total synthesis of spirotryprostatin A (**1**). The overall reaction pathway requires only four steps, yielding a final overall efficiency of 6.2%. However, it is noted that the overall yield is relatively low, and configuration changes occur under alkaline conditions, resulting in the generation of byproducts.

## 3. 1,3-Dipolar Cycloaddition

### 3.1. Williams’s Total Synthesis

**Scheme 7:**
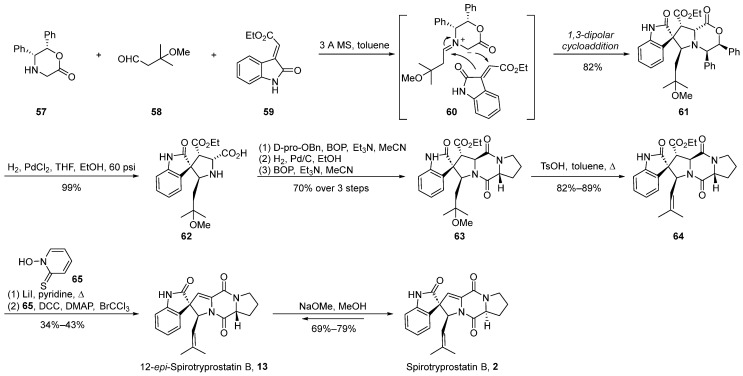
Williams’s total synthesis of spirotryprostatin B via 1,3-dipolar cycloaddition.

The 1,3-dipolar cycloaddition method has proven to be an exceedingly efficacious strategy for constructing helical stereocenters [44,45,46,47]. This approach has been widely applied in the total synthesis of numerous natural products and their analogs [48,49,50,51]. In the year 2000, Williams et al. [52] initially reported the application of the methylimine ylide-mediated 1,3-dipolar cycloaddition method for the total synthesis of spirotryprostatin B (**2**) and 12-*epi*-spirotryprostatin B (**13**). Subsequently, in 2002 [53], further refinements were introduced to enhance the efficacy of this methodology (Scheme 7). The reaction commenced with quinone **57**, aldehyde **58**, and oxindole-based ethyl oxindole-2-carboxylate **59** as substrates. Initially, under the influence of molecular sieves in toluene, quinone **57** and aldehyde **58** underwent condensation, forming imine ylide intermediate **60**. Subsequent to this step, a 1,3-dipolar cycloaddition reaction ensued with ethyl oxindole-2-carboxylate **59**, resulting in the formation of spirocyclic intermediate **61**. The stereochemical assignment of compound **61** was elucidated through single-crystal X-ray crystallography, revealing the authors’ proposition that steric hindrance effected by the isoprene aldehyde precursor favors the adoption of the E-stereoisomer geometry. Consequently, achieving high stereochemical selectivity in spirotryprostatins bearing the isoprene moiety at carbon position C18 was feasible. The ensuing steps involved hydrogenation for benzyl group removal to yield **62**, followed by ring opening of the lactone, amidation, elimination, and Hunsdiecker reaction, ultimately affording 12-*epi*-spirotryprostatin B (**13**). Upon treatment with methoxide in methanol, 12-*epi*-spirotryprostatin B (**13**) underwent isomerization, culminating in the target compound (-)-spirotryprostatin B (**2**). The overall synthetic route encompassed nine steps with a yield of 11%.

**Scheme 8:**
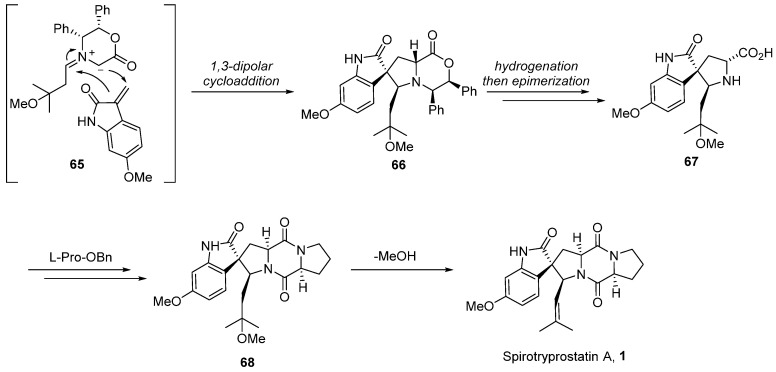
Williams’s total synthesis of spirotryprostatin A *via* 1,3-dipolar cycloaddition.

In 2004, Williams [54,55] extended the application of this methodology to the total synthesis route of spirotryprostatin A (**1**) (Scheme 8). Given the structural disparities between spirotryprostatins A and B, primarily attributed to the methoxy-substituted indole moiety on the benzene ring in spirotryprostatin A and the double bond in the pyrrolidine of spirotryprostatin B, the authors initiated synthesis with indole-2-ketone, possessing the methoxy substituent. A 1,3-dipolar cycloaddition reaction was employed to furnish spiro indoline ketopyrrolidine intermediate **66**, featuring four adjacent chiral centers with methoxy substituents. Subsequent analogous treatment procedures led to the final product, spirotryprostatin A (**1**), with a yield of 2%. The primary challenge in achieving a higher yield was attributed to suboptimal chiral control during the 1,3-dipolar cycloaddition step, resulting in the formation of multiple stereoisomers.

### 3.2. Waldmann’s Total Synthesis

**Scheme 9:**
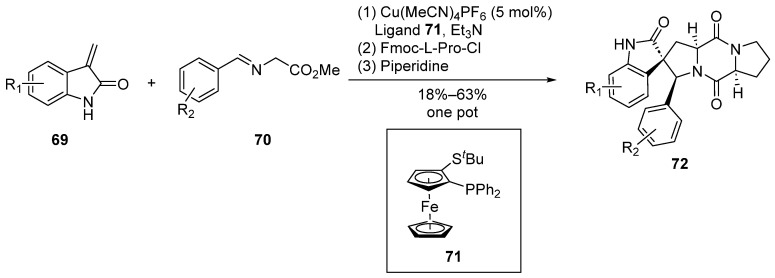
Waldmann’s total synthesis of spirotryprostatin A scaffold via 1,3-dipolar cycloaddition.

In 2011, Waldmann et al. [56] reported, for the first time, a Cu/*N*,*P*-bis(2-methylphenyl)ferrocenyl Lewis acid-catalyzed 1,3-dipolar cycloaddition reaction (Scheme 9). Employing a concise one-pot, three-step methodology, the researchers achieved high enantiomeric selectivity in the synthesis of spirocyclic pyrrolidine–indolone compounds. Subsequently, leveraging this structural motif, a series of derivatives based on the spirotryprostatin A scaffold were successfully synthesized. Notably, this synthetic route demonstrated remarkable enantiomeric selectivity, reaching up to 97%.

### 3.3. Gong’s Total Synthesis

**Scheme 10:**
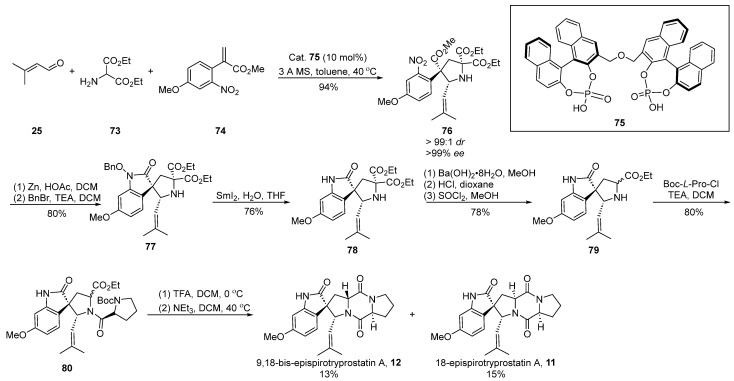
Gong’s total synthesis of spirotryprostatin alkaloids via 1,3-dipolar cycloaddition.

In the same year, Gong [57] introduced a chiral phosphoric acid-catalyzed [3 + 2] cyclization strategy, culminating in the construction of chiral quaternary carbon centers (Scheme 10). This innovative approach facilitated the total synthesis of two distinct enantiomers of spirotryprostatin A. The reaction sequence involved the generation of an imine through the reaction of aldehyde **25** with ethyl aminopropionate **73**, followed by a 1,3-dipolar cycloaddition with methyl acrylate derivative **74** under the catalysis of chiral phosphoric acid **75**. This process not only facilitated the construction of two chiral carbon centers but also resulted in the formation of intermediate **76**. Subsequent zinc powder-mediated cyclization of **76** yielded indole derivative **77**. Compound **77** underwent sequential transformations, including deprotection via samarium diiodide, decarboxylation, and hydrolysis, yielding indole spirocyclic pyrrolidine intermediate **79**. The further condensation of **79** with proline acyl chloride, Boc removal, and closure of the diketopiperazine ring ultimately afforded the target compounds 9,18-*epi*-spirotryprostatin A (**12**) and 18-*epi*-spirotryprostatin A (**11**). Upon conducting activity tests on both non-enantiomeric isomers, it was observed that their activities were comparable to that of spirotryprostatin A (**1**). Consequently, it can be inferred that the configuration at positions C-9 and C-18 in such compounds is not a decisive factor influencing the structure–activity relationship of spirotryprostatin A (**1**). The synthesis involved a total of 10 steps, culminating in the production of two non-enantiomeric isomers, namely 9,18-*epi*-spirotryprostatin A (**12**) and 18-*epi*-spirotryprostatin A (**11**), with overall yields of 4.9% and 5.3%, respectively.

### 3.4. Wang’s Total Synthesis

**Scheme 11:**
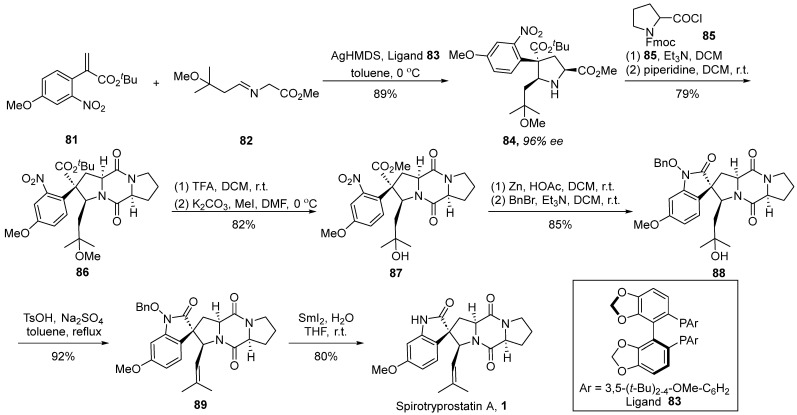
Wang’s total synthesis of spirotryprostatin A via 1,3-dipolar cycloaddition.

In 2023, Wang’s group [58] disclosed an instance of a silver-catalyzed asymmetric [3 + 2] cycloaddition strategy, achieving the total synthesis of spirotryprostatin A (Scheme 11). The authors initiated the synthesis utilizing α-substituted acrylic ester **81** and imine ester **82** as starting materials. Employing AgHMDS and chiral ligand 83 as catalysts facilitated a cyclization process, affording methyl ester derivative **84** with a 96% ee value. Through a coupling reaction with acyl chloride **85**, using the methodology established by the Danishefsky group, the formation of fused-ring compound **86** was achieved. Subsequently, intermediate **86** underwent demethoxylation and methylation under the influence of trifluoroacetic acid, yielding intermediate **87**. The reduction of the nitro group followed by intramolecular cyclization and benzyl protection afforded pivotal spirocyclic oxindole intermediate **89**. Further transformations involving hydroxyl elimination and deprotection culminated in the final product, spirotryprostatin A (**1**). The synthetic route encompassed a total of six steps with a yield of 36%.

## 4. Metal Catalysis

### 4.1. Heck Coupling

#### 4.1.1. Overman’s Total Synthesis

**Scheme 12:**
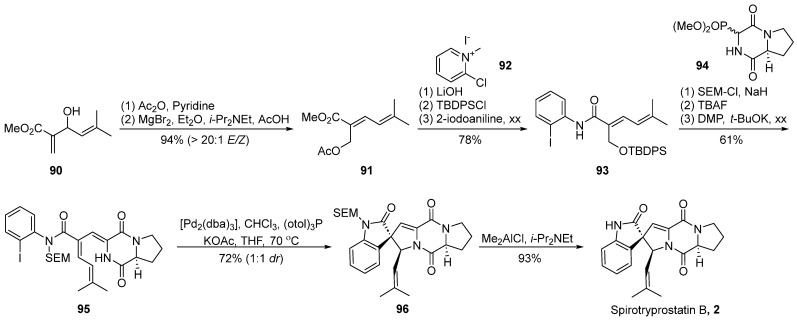
Overman’s total synthesis of spirotryprostatin B *via* Heck coupling.

In the year 2000, Overman et al. [59] achieved the total synthesis of spirotryprostatin B (**2**) through the strategic implementation of a Heck reaction-mediated cyclization to construct the oxygenated spirocyclic framework (Scheme 12). Diverging from the conventional synthetic approaches to spirotryprostatin-type compounds, which typically involve the initial construction of the spirocyclic oxindole moiety, Overman introduced a pioneering strategy wherein the diketopiperazine scaffold is first assembled, followed by the subsequent synthesis of the spiroatom. A pivotal aspect of this synthetic pathway involves the utilization of an intramolecular asymmetric Heck reaction and the dual capture of a π-allyl palladium intermediate by the adjacent amide nitrogen atom within the diketopiperazine ring. This intermediate is subsequently captured by a trans-selective nucleophilic reagent, concurrently establishing chiral centers at the C3 and C18 positions. The reaction commenced with the commercially available substrate allylic alcohol **90**. Initially, an oxygen-induced Claisen rearrangement yielded dienoic ester **91**. The subsequent hydrolysis of the two ester groups on **91**, followed by TBDPS protection and condensation with ortho-iodoaniline, furnished compound **93**. Nitrogen protection of the amide on **93** was accomplished using 2-(trimethylsilyl)ethoxymethyl (SEM), followed by the removal of the TBDPS-protecting group from the hydroxyl moiety. Oxidation with DMP yielded the intermediate aldehyde, which underwent a Horner–Wadsworth–Emmons reaction with phosphonate **94**, culminating in the formation of compound 95. The final steps involved a Heck reaction for cyclization, leading to the construction of the oxindole and spirocyclic oxindole compound **96**, concurrently generating an equivalent non-enantiomeric isomer. The demethylation of the target configurational intermediate **96** and subsequent steps resulted in the total synthesis of the natural product spirotryprostatin B (**2**) in 10 steps, achieving a yield of 9%.

#### 4.1.2. Fukuyama’s Total Synthesis

**Scheme 13:**
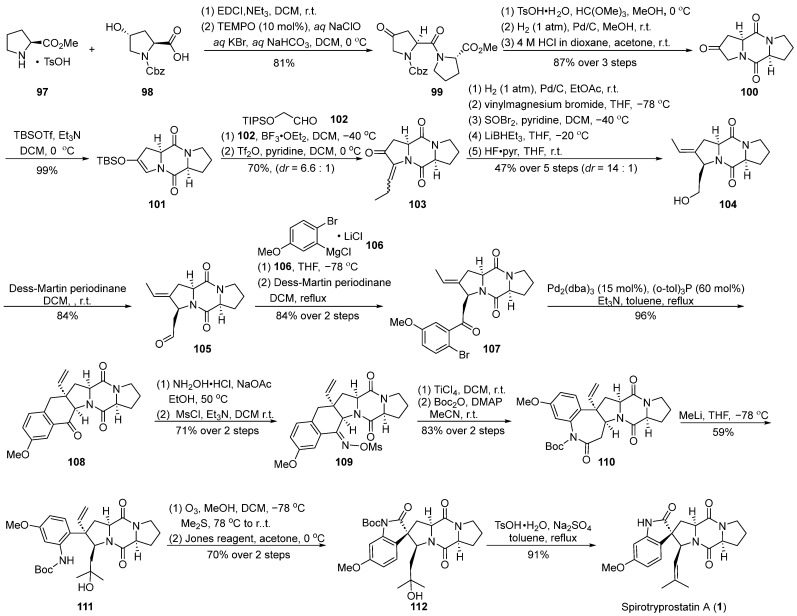
Fukuyama’s total synthesis of spirotryprostatin A via Heck coupling.

In 2014, Fukuyama’s group [60] presented a comprehensive synthesis strategy for spirotryprostatin A (**1**), with a pivotal molecular intramolecular Heck reaction (Scheme 13). Commencing with straightforward *L*-proline methyl ester salt **97** and 4-hydroxy-*L*-proline **98**, the synthesis initiated with a condensation reaction, followed by oxidation using 2,2,6,6-tetramethylpiperidin-1-oxyl (TEMPO) to yield the highly unstable amide intermediate **99**. Recognizing the instability of **99** and its potential impact on yield, a protective measure involved the initial carbonyl protection of the keto group in **99** to form a dimethyl ketal. The subsequent hydrogenation of the formate benzyl ester (Cbz) group triggered an intramolecular cyclization, resulting in the formation of the diketopiperazine intermediate, which, upon acidic hydrolysis, yielded the diketopiperazine intermediate **100**. The treatment of ketone **100** with a combination of trifluoromethanesulfonic anhydride (TBSOTf) and triethylamine provided the desired intermediate **101**, featuring an unsaturated pyrrolidine structure. The resulting product exhibited high specificity, achieving a remarkable yield of 99%. To introduce an alkyl side chain at the C-18 position of the final product, the Mukaiyama hydroformylation reaction was employed between **101** and trimethylsilyloxyacetaldehyde **102**. Subsequent dehydration with trifluoromethanesulfonyl anhydride resulted in the formation of the unsaturated ketone **103** in a mixture of *E-Z* isomers (6.6:1). Utilizing palladium catalysis, the hydrogenation of **103** was accomplished, followed by a consecutive three-step reduction–rearrangement process, and deprotection, selectively converting it into intermediate **104**. Subsequently, the oxidation of the secondary alcohol to an aldehyde was achieved using a Dess–Martin reagent, and further nucleophilic addition with a Grignard reagent produced an intermediate, which, upon subsequent oxidation with the Dess–Martin reagent, led to the formation of ketone intermediate **107**. Under palladium catalysis, intermediate **107** underwent an intramolecular Heck coupling cyclization reaction, resulting in the formation of tetrahydronaphthone **108**. The introduction of the nitrogen moiety was achieved through hydroxylamine hydrochloride, generating the *E*-oxime **109** because of the hinderance of the Ph-ring. Subsequent Beckmann rearrangement, Boc protection, and ring opening with methyl lithium yielded the tertiary alcohol intermediate **111**. Ozone decomposition followed by an oxidation step resulted in the formation of the spirocyclic oxindole ketone intermediate **112**. Finally, through dehydration and deprotection steps, the ultimate product, spirotryprostatin A (**1**), was obtained. The synthetic pathway involved a total of 25 steps with an overall yield of 3.4%. Despite the relatively high yields at each step, the extended length of the synthetic route contributed to a lower final yield.

### 4.2. Metal Olefination

#### 4.2.1. Fuji’s Total Synthesis

**Scheme 14:**
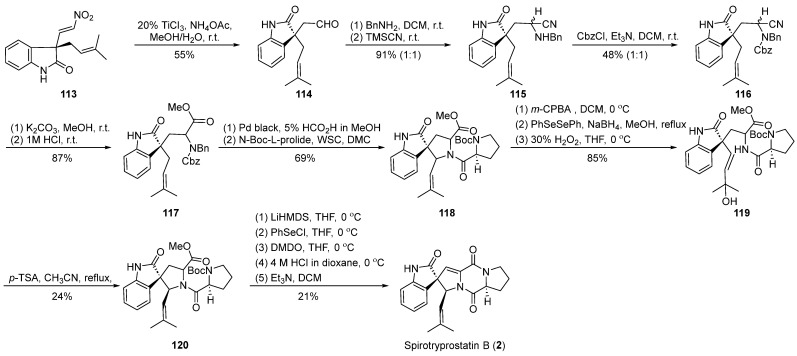
Fuji’s total synthesis of spirotryprostatin B via nitroalkene cyclization.

In the year 2002, Fuji et al. [61] utilized nitroalkene cyclization as a pivotal step in the synthesis of spirotryprostatin B (**2**) (Scheme 14). Their strategy for constructing the spirocyclic quaternary carbon was guided by the formation of a chiral auxiliary, whereby the resulting intermediate underwent functional group transformations, introducing distinct fragments and ultimately yielding the cyclization precursor **113**. Under the influence of titanium trichloride and ammonium acetate, precursor **113** underwent nitroalkene transformation to yield aldehyde **114**. Subsequent steps, including Strecker reaction, Cbz protection, and cyanide hydrolysis, led to the formation of a benzylamine intermediate **117** containing an ester moiety. Compound **117** was further condensed with protected *L*-proline to generate amide intermediate **118**. Successive transformations involving epoxidation, phenylselenol addition, and oxidative cleavage resulted in the conversion to tertiary alcohol intermediate **119**. The treatment of intermediate **119** with *p*-toluenesulfonic acid produced the anticipated spirocyclic oxindole pyrrolidine intermediate **120** with a yield of 24% and the concomitant formation of an isomer. Employing the methodology reported by Danishefsky et al., the treatment of **120** yielded the target product spirotryprostatin B (**2**), following a 16-step synthetic route with an overall yield of 0.6%, accompanied by a notable presence of byproducts.

#### 4.2.2. Trost’s Total Synthesis

**Scheme 15:**
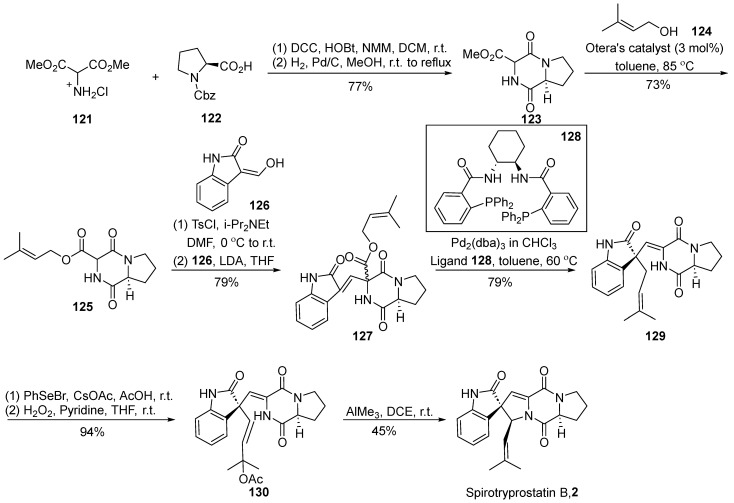
Trost’s total synthesis of spirotryprostatin B via palladium-catalyzed asymmetric isoprene functionalization.

In 2007, Trost et al. [62] disclosed a comprehensive synthesis route for spirotryprostatin B (**2**) utilizing an efficient palladium-catalyzed asymmetric isoprene functionalization (Scheme 15). A pivotal aspect of this synthetic pathway involves the non-enantioselective isoprenylation reaction under palladium catalysis to construct the spirocyclic carbon stereocenter. Commencing with Cbz-protected *L*-proline **122** and hydrochloride salt of 2-amino-propane-dicarboxylic acid **121**, the reaction involved an initial sequence of condensation, hydrogenation, deprotection, and subsequent condensation to yield intermediate **123**. Under the catalysis of the Otera catalyst, intermediate **123** underwent alkylation to generate the diketopiperazine compound **125** bearing an isoprenyl side chain. The coupling of intermediate **125** with oxindole **126**, facilitated by a one-pot strategy, produced the nucleophilic precursor compound **127**. Subsequent decarboxylative isoprenyl rearrangement occurred under the joint action of a palladium catalyst and ligand **128**, yielding the crucial intermediate **129**. The treatment of intermediate **129** with PhSeOAc, followed by oxidative elimination using hydrogen peroxide, led to the formation of vinyl acetate **130**. Ultimately, the target product was obtained through cyclization under the influence of trimethylaluminum. The entire synthetic route comprised eight steps, achieving the total synthesis of spirotryprostatin B (**2**) with a yield of 13.7%.

### 4.3. Cascade Reactions

#### 4.3.1. Procter’s Total Synthesis

**Scheme 16:**
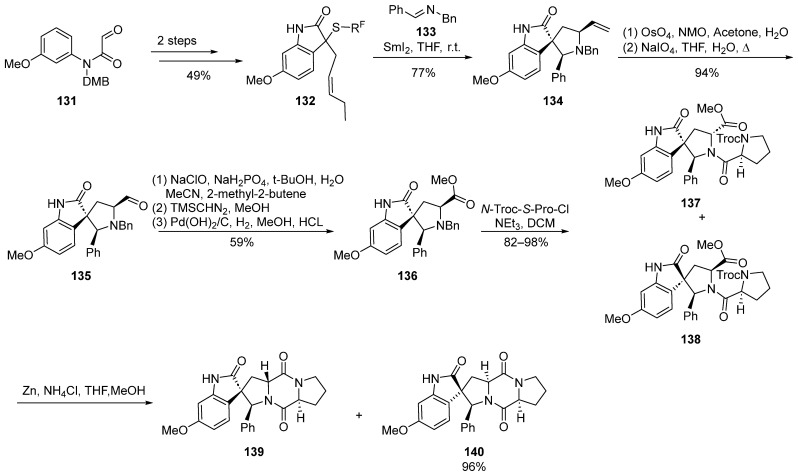
Procter’s total synthesis of spirotryprostatin alkaloids via iodine-induced cascade reactions.

In 2011, Procter et al. [34] documented a cascade reaction triggered by iodine-induced ring formation in the synthesis of spirocyclic indolinone compounds (Scheme 16). Through extensive exploration, they delineated a concise synthetic route for the total synthesis of 18-phenyl-substituted spirotryprostatin A derivatives, spanning a comprehensive sequence of 10 steps with an overall yield ranging between 13.7% and 16.3%. The reaction commenced with amide compound **131** as the starting material, undergoing a two-step transformation to yield the indolone intermediate **132**. Subsequently, a tandem reaction employing samarium iodide catalysis and imine **133** led to the formation of the spirocyclic pyrrolidine intermediate **134**. The subsequent oxidative cleavage of the terminal alkene in **134** furnished aldehyde intermediate **135**. Sequential oxidation, esterification, and debenzylation reactions yielded methyl ester intermediate **136**, which, upon condensation with proline acyl chloride, generated the mixture of compounds **137** and **138**. The subsequent deprotection of the Troc-protecting group initiated a cyclization reaction, affording two derivatives of spirotryprostatin A, **139** and **140**, both featuring an 18-phenyl substitution. In the initial stages of biological activity assessment, the authors observed that compound **140** exhibited a level of bioactivity comparable to that of spirotryprostatin A (**1**). This finding may provide valuable insights into the structure–activity relationships governing the biological activities of spirotryprostatin-like compounds, thereby serving as a guiding reference for further investigations in this domain.

#### 4.3.2. Shen’s Total Synthesis

**Scheme 17:**
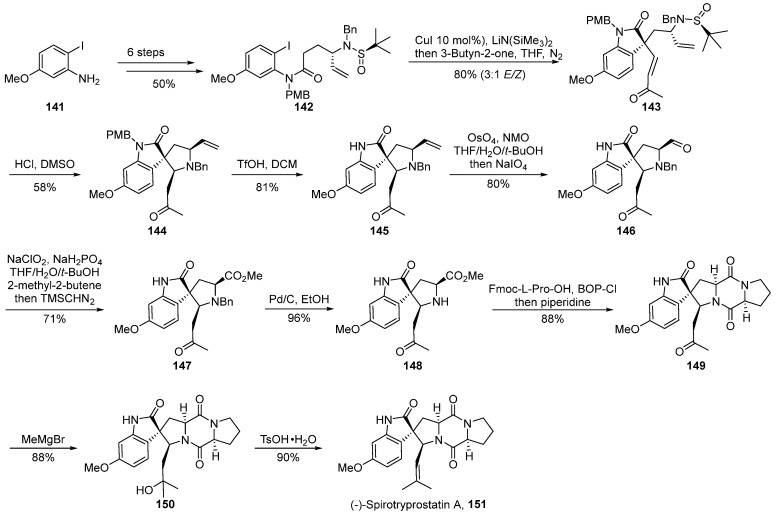
Shen’s total synthesis of spirotryprostatin alkaloids via copper-catalyzed cascade reactions.

In the year 2022, Shen’s group [63] documented a strategy involving chiral auxiliary-mediated, copper-catalyzed cascade reactions for the introduction of quaternary carbon stereocenters and nitrogen-containing Michael cascade reactions (Scheme 17). This approach facilitated the asymmetric total synthesis of (-)-spirotryprostatin A **151**, thereby constructing the spirocyclic pyrrolidine-indole framework. The reaction commenced with 2-iodo-5-methoxyaniline **141** as the initial substrate and underwent a concise six-step transformation to yield the ortho-iodoaniline derivative **142**. Subsequently, a copper-catalyzed tandem reaction with an alkyne ketone was employed, introducing a quaternary carbon stereocenter and yielding the pivotal spirocyclic oxidized indole intermediate **143**. Following this, under acidic conditions, intermediate **143** underwent removal of the chiral auxiliary and intramolecular cascade aza-Michael reaction, yielding intermediate **144**. The treatment of **144** with triflic anhydride removed the protective group, and the subsequent oxidation of the olefin under the influence of osmium tetroxide/sodium periodate produced aldehyde **146**. Aldehyde **146** underwent Pinnick oxidation in a buffered solution, followed by esterification with (trimethylsilyl) diazomethane to afford methyl ester intermediate **147**. Intermediate **147**, under palladium–carbon catalysis, underwent deprotection of the silyl group, followed by a ring-closing reaction using the improved strategy by the Danishefsky research group, leading to intermediate **149**. Finally, a formaldehyde reagent addition and subsequent dehydration reactions were employed to achieve the total synthesis of the target compound (-)-spirotryprostatin A **151**. The entire synthetic pathway encompassed 15 steps, yielding the final product at an overall yield of 7.4%.

### 4.4. MgI_2_-Catalyzed Ring Expansion

#### Carreira’s Total Synthesis

**Scheme 18:**
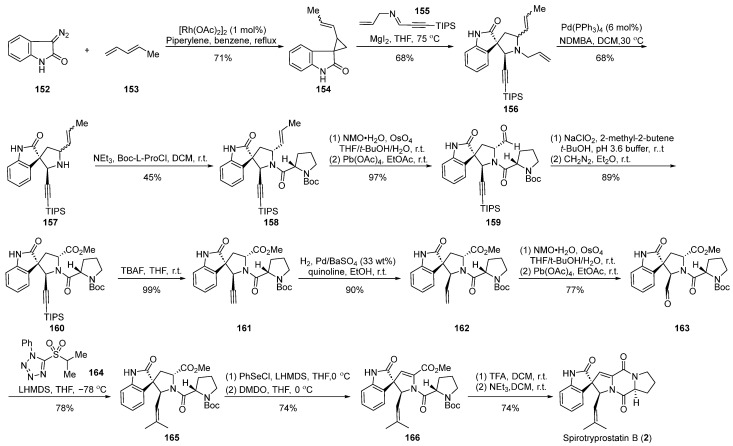
Carreira’s total synthesis of spirotryprostatin B via MgI_2_-catalyzed ring expansion.

In 2003, the Carreira group reported [64] the application of magnesium iodide-catalyzed cyclization in the total synthesis of spirotryprostatin B (**2**) (Scheme 18). The reaction commenced with diazoindolone **152** as the starting material, which, under rhodium catalysis, underwent a cyclopropanation reaction with pentadiene **153**, affording the indolone cyclopropane intermediate **154**. Intermediate **154** and imine **155**, through a magnesium iodide-catalyzed [1 + 3] ring addition and ring expansion reaction, yielded the spirocyclic intermediate **156**. The subsequent palladium-catalyzed removal of the allyl group produced intermediate **157**. A Schotten–Baumann reaction with *N*-Boc-*L*-proline acyl chloride introduced the proline five-membered ring, followed by a series of oxidation reactions to obtain aldehyde **159**. Aldehyde **159**, subjected to Pinnick oxidation, TIPS deprotection, and hydrogenation, yielded compound **162**. Further oxidation and olefination led to the spirocyclic pyrrolidine intermediate **165**, which, employing Danishefsky’s methodology, underwent a PhSeCl-mediated elimination reaction followed by Boc-deprotection and closure of the diketopiperazine ring to ultimately obtain the target product spirotryprostatin B (**2**). The synthetic route comprised 19 steps, with intricacies arising from isomer separations and demanding reaction conditions, contributing to a relatively low overall yield of 5.5%.

## 5. Conclusions

To date, various synthetic methodologies have been developed for the total synthesis of spirotryprostatin alkaloids, resulting in a diverse array of spirotryprostatin compounds. However, certain approaches, such as oxidative rearrangement, have encountered challenges related to selectivity. Although some methods, such as copper- and silver-catalyzed 1,3-dipolar cycloaddition reactions, show promising potential for asymmetric synthesis, the necessity for the preparation of highly effective chiral ligands to control stereochemical issues poses a significant hurdle to achieving an efficient and concise process, requiring prolonged development efforts. Other approaches utilizing palladium, rhodium, and other metal-catalyzed methods, while capable of stereoselectively synthesizing spirocyclic oxindole diketopiperazines, often involve complex routes, low yields, and the use of expensive noble metals as catalysts, hindering their broader applicability. With the increasing discovery and isolation of spirocyclic oxindole diketopiperazine alkaloids, a continual emergence of highly selective, efficient, and concise synthetic methods is anticipated. Given the notable biological activity of spirotryprostatin compounds, an expanding repertoire of such compounds is being synthesized and applied in clinical practice. It is anticipated that, in the near future, a cohort of new drugs based on the spirotryprostatin scaffold will emerge in the field of medicine, serving as representative products in the development of novel anti-tumor drugs.

## Figures and Tables

**Table 1:** Summary of total syntheses of spirotryprostatin alkaloids.

Groups	Year	Key Mechanism	Steps and Yields
Danishefsky	1998	oxidative rearrangement	8 steps with yield of 6.5%
Granesan	2000		5 steps with yield of 2–6%
Zhang	2019		11 steps with yield of 20%
Danishefsky	2000	Mannich reaction	8 steps with yield of 4.6%
Horne	2004		7 steps with yield of 4.9%
	2004		6 steps with yield of 6.2%
William	2002	1,3-dipolar cycloaddition	9 steps with yield of 11%
Williams	2004		4 steps with yield of 2%
Waldmann	2011		1 step with yield of 97%
Gong	2011		10 steps with yield of 4.9% and 5.3%
Wang	2023		6 steps with yield of 36%
Overman	2000	heck coupling	10 steps with yield of 9%
Fukuyama	2014		25 steps with yield of 3.4%
Fuji	2002	nitroalkene cyclization	16 steps with yield of 0.6%
Trost	2007	palladium-catalyzed asymmetric isoprene functionalization	8 steps with yield of 13.7%
Procter	2011	cascade reaction triggered by iodine-induced ring formation	10 steps with yield of 13.7%
Shen	2022	copper-catalyzed cascade reactions	15 steps with yield of 7.4%
